# Inhibitory Effect of Resveratrol against Duck Enteritis Virus *In Vitro*

**DOI:** 10.1371/journal.pone.0065213

**Published:** 2013-06-11

**Authors:** Jiao Xu, Zhongqiong Yin, Li Li, Anchun Cheng, Renyong Jia, Xu Song, Hongke Lu, Shujun Dai, Cheng Lv, Xiaoxia Liang, Changliang He, Ling Zhao, Gang Su, Gang Ye, Fei Shi

**Affiliations:** 1 College of Veterinary Medicine, Sichuan Agricutural University, Ya'an, China; 2 Institute of Preventive Veterinary Medicine, Sichuan Agricutural University, Chengdu, China; Northeast Agricultural University, China

## Abstract

Duck viral enteritis (DVE) is an acute, contagious herpesvirus infection of ducks, geese, and swans of all ages and species. This disease has been responsible for significant economic losses in domestic and wild waterfowl as a result of mortality, and decreased egg production. Resveratrol is a naturally occurring phytoalexin in specific plants and exhibits inhibitory activity against many kinds of virus. In this paper, resveratrol was found to inhibit duck enteritis virus (DEV) replication in a dose-dependent manner, with a 50% inhibition concentration of 3.85 μg/mL. The inhibition in virus multiplication in the presence of resveratrol was not attributed to direct inactivation or inhibition of virus attachment to the host cells, but to the inhibition of viral multiplication in host cells. The assay of the time of addition limited the drug effect during the first 8 h of infection. This conclusion was supported by the ultrastructure images of the early stage of DEV infection, which showed that the replication of virus nucleic acid and the formation of the capsid in the cell nucleus were suppressed. In the indirect immunofluorescence assay, proteins expression in DEV infected duck embryo fibroblasts (DEFs) within 24 h post-infection (p.i.) was also effectively suppressed by resveratrol. In summary, the resveratrol has a good activity against DEV infection *in vitro*, which could be attributed to that fact that several essential immediate early viral proteins for virus replication were impacted by resveratrol.

## Introduction

Duck enteritis virus (DEV), a member of the alphaherpesvirinae subfamily of the herpesviridae, is an acute, contagious virus infection that naturally occurs in ducks, geese and swans, which are all members of the family Anatidae of the order Anseriformes [1]. In susceptible flocks, this disease can be transmitted by direct contact or indirectly through environmental contamination [2],[3]. It causes significant economic losses in domestic and wild waterfowl as a result of mortality, and decreased egg production [4]. As a major method for controlling this disease, the expanded use of vaccines could bring considerable benefit in reducing infected ducks morbidity and mortality, but sometimes infection could still not be prevented [5],[6],[7].

Natural medications are attracting increasing interest in the development of potential anti-viral drugs. We have been engaged in research on thistopic for a long period. Many herbs were investigated for their antiviral activities in our laboratory [8]. Resveratrol, a non-flavonoid polyphenol compound, is a naturally occurring phytoalexin produced by several higher plants such as mulberries, peanuts and grapes in response to stress or injury induced by environmental hazards [9]. This compound has been shown to possess high bioactivities against many diseases, such as cancers, cardiovascular diseases, inflammation, diabetes and viral diseases. In several studies, resveratrol was found to increase the potency of several antiretroviral drugs synergistically against human immunodeficiency virus (HIV) [10] and herpes simplex viruses (HSV) [11]. Resveratrol was also found to be effective in treating influenza virus and severe acute respiratory syndrome (SARA) [12]. In an *in vivo* study, 25% resveratrol cream could significantly inhibit the development of HSV-1 induced skin lesions on the abraded epidermis of SKH1 mice, and the therapeutical effect is the same as that of 5% acyclovir cream [13].

As a potential antiviral agent against several species of virus, especially for the good treatment of HSV, in this paper, resveratrol was tested for antiviral activity against DEV, which is also a member of the alphaherpesvirinae subfamily of the herpesviridae. This paper aims to evaluate the antiviral properties of resveratrol and conduct a preliminary study on its antiviral mechanisms against DEV.

## Materials and Methods

### Compounds and reagents

Resveratrol was bought from XI'AN CENTER BIO-TECH CO., LTD. (Xi'an, P.R. China) with a purity of 98%, which was dissolved in dimethylsulfoxide (DMSO) before use (25 mg/mL) and filtered using a 0.22 μm millipore filter to eliminate bacteria. Rabbit anti-DEV antibody was provided by the Institute of Preventive Veterinary Medicine, Sichuan Agricultural University (Chengdu, P.R. China). FITC-labeled goat anti-rabbit antibody was obtained from Boster (Wuhan, P.R. China).

### Cells and virus

Duck embryo fibroblasts (DEFs) were propagated in minimal essential medium (MEM) (Gibco-BRL, Grand Island, NY, USA) supplemented with 10% calf serum (CS) (Gibco-BRL, Grand Island, NY, USA). All cell cultures were incubated at 37°C in a humidified atmosphere supplied with 5% CO_2_. The DEV CH virulent strain (DEV-CHv) -[14] was kindly provided by Prof. An-chun Chen of the Institute of Preventive Veterinary Medicine, Sichuan Agricultural University (Chengdu, P.R. China).

### Cytotoxicity assay

The cytotoxic effect of resveratrol on DEFs monolayers were determined by quantifying the cell viability using an MTT (3-[4.5-dimethylthiazol-2-yl]-2,5-diphenyl tetrazolium bromide; Sigma, USA) assay [15],[16],[17]. In the assay, DEFs in 96-well plates were exposed to different concentrations of compounds in triplicate, at 100 μL per well. The test samples were suspended with MEM supplemented with 2% CS. The dilution medium without the sample was used as control. After 3 d of incubation at 37°C in humidified 5% CO_2_, 10 μL of the MTT solution [5 mg/ml in phosphate buffered saline (PBS)] was added to each well. After 4 h of incubation at 37°C, the supernatant was removed and 100 μL of DMSO was added to each well. After vigorous shaking, the absorbance values were measured using a microplate reader (Bio-Rad, USA) at 570 nm. The 50% half cell toxicity (CC_50_) value was calculated as the extract concentration necessary to reduce cell viability by 50%. The maximal non-cytotoxic concentration (MNCC) was defined as the maximal concentration of the sample that did not exert a significant cytotoxic effect (*P*<0.01) based on the OD values.

### Antiviral activity assay

A 30 μL suspension containing 100 times 50% tissue culture infectious doses (TCID_50_) of virus and a serial two-fold dilution of the resveratrol solution were added on monolayers of DEFs in 96-well plates, which were incubated at 37°C for 1 h to allow attachment. Thereafter, the medium was aspirated from the well to remove the unabsorbed virus. The cell monolayers were then washed with PBS and medium only with dilutions of the test samples were added to the plates respectively. The concentrations of the test samples were equal or less than MNCC. The virus-infected cells without drug treatment and the un-infected cells were used as the controls. The study was performed in triplicate and all plates were incubated at 37°C in a humidified 5% CO_2_ and examined daily under inverted microscope. The virus-induced cytopathic effect (CPE) of the tests was observed under light microscopy and was compared with the virus and cell controls. When the untreated virus-infected cells showed 4+CPE, The MTT assay described above was performed. The inhibition concentration of 50% (IC_50_), which was estimated from the plots of the data, was the concentration that reduced 50% of dead cells relative to that of virus control.

Furthermore, the antiviral activity was also evaluated by the virus yield reduction assay. The DEFs grown in 96-well plates infected with DEV (100TCID_50_) were incubated in the presence or absence of resveratrol according to the method described above. After incubation at 37°C in humidified 5% CO_2_ for 72 h, the TCID_50_ of the cell supernatant of each well was determined in DEFs with 10-fold serially diluted virus incubated at 37°C in humidified 5% CO_2_ for 72 h. Virus titers in TCID_50_/50 μL were determined according to the Reed and Muench method [18].

### Influence of resveratrol on the viral growth curve

Copies of viral genomes of DEV at the different treatments of the drug were detected using the real-time fluorescence quantification PCR (FQ-PCR) method. Total DNA was extracted from DEV infected DEFs using a DNAiso Reagent (TaKaRa, Japan), and was analyzed using Premix Ex Taq^TM^ (Probe qPCR) (TaKaRa, Japan). The primers and probe were designed using the Primer software (Version 5.0; Primer Biosoft International, Palo Alto, CA). The upstream and downstream primer sequences were 5′-CCCAAACACGAAACATGC-3′ and 5′- TGTCCGGTTACAATATCGTT -3′, respectively, which were used to amplify a 123-bp fragment of the DNA polymerase gene (GenBank accession no. JQ647509.1) of DEV. A 22-bp probe (5′-CTCCTTTGTTCATCGCCCCGTA-3′) complementary to an internal region between two primers was selected and labeled with carboxy-fluorescein at the 5′ end and with carboxytetramethylrhodamine at the -3′ end. The primers and probe were synthesized by Invitrogen Trading CO., LTD. (Shanghai China).

The real-time FQ-PCR conditions consisted of one cycle of 30 s at 95°C followed by 40 cycles of 5 s at 95°C and 30 s at 57.5°C. Data analysis was performed using Bio-Rad CFX96^TM^ Manager software. The number of target copies in the reaction was deduced from the threshold cycle (*C*_T_) values corresponding to the *C*_T_ values of the serial 10-fold dilutions of standard DNA samples.

The DEFs grown in 6-well plates were first infected with DVEV at an MOI of 1 and resveratrol solution for 1 h at 4°C. Resveratrol or a medium without the test drug were added to the cultures after removal of the inoculums. The total DNA was extracted from DEV-infected DEFs at the indicated time points (2, 4, 6, 8, 10, 12, 24, 48, and 72 h post-infection [p.i.]) and the copies of DEV were detected by the real-time FQ-PCR assay.

### The mode of action assay

#### Virus inactivation assay

Equal volumes of resveratrol solution and a concentrated virus suspension were placed in a tube for 1 h at 37°C. The mixture without the test sample was used as control. The infectious virus that remained in the drug was diluted 100-fold (MOI = 0.1) and then added in cell monolayers, incubating for 1 h at 37°C. MEM supplemented with 2% CS was added to the cultures after removal of the virus inoculums. After incubation for 48 h, the total DNA was extracted from DEV infected DEFs and copies of DEV were detected by the real-time FQ-PCR assay.

#### Inhibition of virus attachment

Equal volumes of the resveratrol solutions and a virus suspension (MOI = 0.1) were placed in a tube and the mixtures were immediately poured on cell monolayers, and then incubated for 1 h °C at 37°C, with or without the test drug. MEM supplemented with 2% CS was added to the cultures after removal of virus inoculums. After incubation for 48 h, the total DNA was extracted from DEV infected DEFs, and copies of DEV were detected by the real-time FQ-PCR assay.

#### Pre-treatment assay

Resveratrol solutions were added in cell monolayers and incubated at 37°C for 1 h, after which the samples were removed. The cell monolayers were then incubated with DEV at an MOI of 0.1 for 1 h at 37°C. MEM supplemented with 2% CS was added to the cultures after removal of the virus inoculums. After incubation for 48 h, the total DNA was extracted from DEV infected DEFs, and copies of DEV were detected by the real-time FQ-PCR assay.

#### Intracellular inhibition assay

The cell monolayers were infected with DEV at an MOI of 0.1 for 1 h at 37°C. The cell monolayers were then washed with PBS and overlaid with resveratrol solutions. The wells overlaid with MEM without test sample were used as the control. After incubation for 48 h, the total DNA was extracted from DEV infected DEFs and copies of DEV were detected by the real-time FQ-PCR assay.

### Influence of treatment time on antiviral activity

Time course analysis was performed as follows: DEFs grown in 96-well plates were fitst infected with 100TCID_50_ for 1 h at 37°C. Resveratrol was added to the cultures after removal of the virus inoculum at the indicated time points (0, 2, 4, 6, and 8 h p.i.). After incubation for 24 h, antiviral activity was evaluated by the virus yield reduction assay.

### Transmission electron microscopy (TEM) assay

The DEFs grown in coverslips infected with DEV (MOI  = 0.1) were incubated in the presence or absence of resveratrol according to the method used for the antiviral activity assay. At 48 h p.i., the monolayers were washed and subsequently scraped away by using a cell scraper (Nunc, USA). After centrifugation at 1200 g for 5 min, the cells were collected and then fixed with 2.5% glutaraldehyde for 2 h at 4°C and 1% osmium tetroxide for 2 h at 4°C. Prior to infiltrating and embedding, the cells were dehydrated with a graded acetone series. Ultrathin (100 nm, Leica UC6), longitudinal sections were then cut and stained with uranyl acetate and lead citrate, and examined using a Tecnai G2 F20 electron microscope (FEI, USA).

### Indirect immunofluorescence assay

The DEFs grown in coverslips infected with DEV (MOI  = 0.1) were incubated in the presence or absence of resveratrol according to the method used for the antiviral activity assay. At 24 h p.i., the cells were washed with cold PBS and fixed in 4% paraformaldehyde for 30 min at room temperature. The cells were then permeabilized using 1% (v/v) Triton X-100 in PBS for 20 min before incubation with 10% goat serum/PBS for 45 min at 37°C. The cells were washed and incubated with rabbit anti-DEV antibody overnight at 4°C. After washing, the cells were incubated with FITC-labeled goat anti-rabbit antibody for 1 h at 37°C. Finally, cells were washed and directly observed using a fluorescence microscope (Eclipse 80i, Nikon, Japan).

### Statistics

All data are representative of at least three independent experiments. Data are presented as mean ± SD. Statistical significance was calculated using the SPSS 17.0 software.

## Results

### Antiviral activity of resveratrol

**Figure 1:**
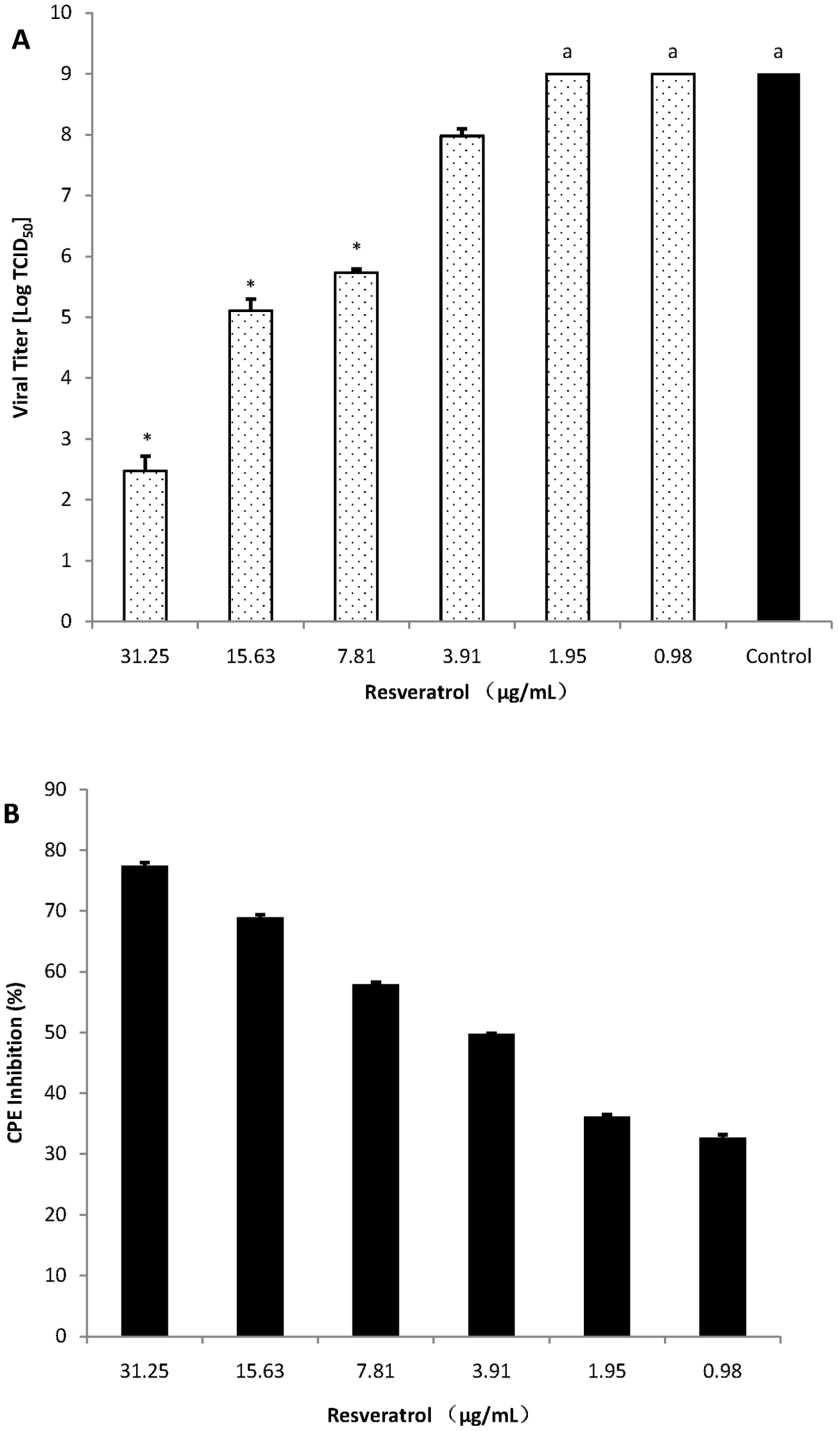
Viral titers of DEV at growing conditions with different concentrations of resveratrol. (A) DEFs were infected with DEV (TCID_50_) and then treated with resveratrol. After incubation for 72 h, the supernatants were collected and analyzed for TCID_50_ values. Values are means ± SD (n = 3). Significance: *P<0.01 vs. non drug treated control group. ^a^ TCID_50_ >9. (B) DEFs were first infected with DEV (TCID_50_) and then treated with resveratrol. After incubation for 72 h, MTT assay was performed, and the results were expressed as percent of inhibition in drug-treated cultures compared with untreated group. Values are means ± SD (n = 3).

Resveratrol was assayed for its capability to inhibit DEV multiplication *in vitro* via CPE, MTT and TCID_50_ assays, as previously described. DEFs were first infected with DEV andthen treated with resveratrol at the indicated concentrations after removal of the virus inoculum. After 72 h, cell viability was evaluated via MTT assay and virus titers of the culture media were determined via the yield reduction assay. The IC_50_ of resveratrol for CPE reduction was approximately 3.85 μg/mL, and the selectivity index (CC_50_/IC_50_) was approximately 15.86 (Table 1). In the virus yield reduction assay, the resveratrol could significantly reduce the virus titer of DEV and the viral titer with different concentrations of resveratrol was does-dependent (Fig. 1A). The CPE inhibitions also showed a does-dependent characteristic, where values increased with increasing resveratrol concentrations. The inhibition raito was >75% at 31.25 μg/mL(Fig. 1B).

**Table 1:** Inhibition effects of resveratrol on DEV *in vitro* a.

Compounds	IC_50_(μg/mL)^b^	CC_50_(μg/mL)^c^	SI^d^
Resveratrol	3.85±0.08	61.07±0.24	15.86

a The inhibition effects on DEV were evaluated by MTT assay.

b Inhibition concentration 50% (IC_50_): concentration required to inhibit DEV at 72 h post-infection by 50%.(n = 3).

c Cytotoxic concentration 50% (CC_50_) concentration required to reduce cell viability by 50%. (n = 3).

d SI: Selectivity index is defined as the radio of CC_50_ to IC_50_.

### Influence of resveratrol on the viral growth curve

**Figure 2:**
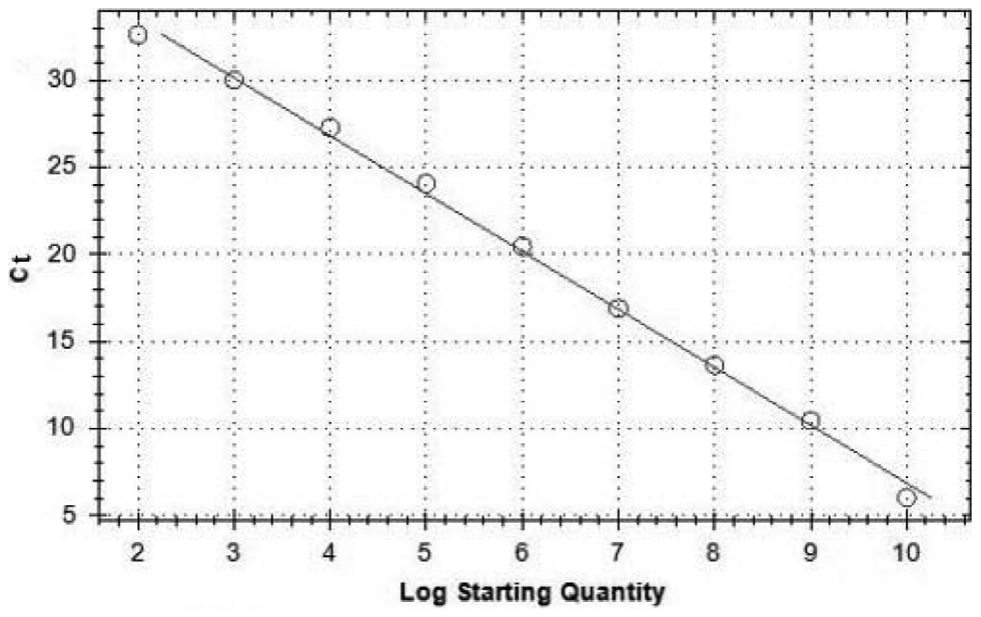
Standard curve of real-time FQ-PCR amplification. Serial 10-fold dilutions of standard DEV DNA from 5.25×10^10^ to 10^2^ copies were amplified in this process. Amplification efficiency (E) was 99.8%. The standard curve equations was *C*t = −3.328×lg [virus copies/5.25]+40.138 (R^2^ = 0.997).

Mechanistic studies were performed at a resveratrol concentration of 31.25 μg/mL in order to assess a high inhibitory effect in all assays that were designed to elucidate the antiviral characteristics of this compound. DEFs were first infected with DEV (MOI = 1) and resveratrol solution for 1 h at 37°C. The DEV proliferated in the DEFs in the presence or absence of reveratrol within 72 h. Total DNA was extracted at the indicated time points (2, 4, 6, 8, 10, 12, 24, 48, and 72 h p.i.) and the copies of DEV were detected by the real-time FQ-PCR assay. The copies of DEV were calculated according to the standard curve equations: *C*t = −3.328×lg [virus copies/5.25]+40.138 (R^2^ = 0.997) (Fig. 2).

**Figure 3:**
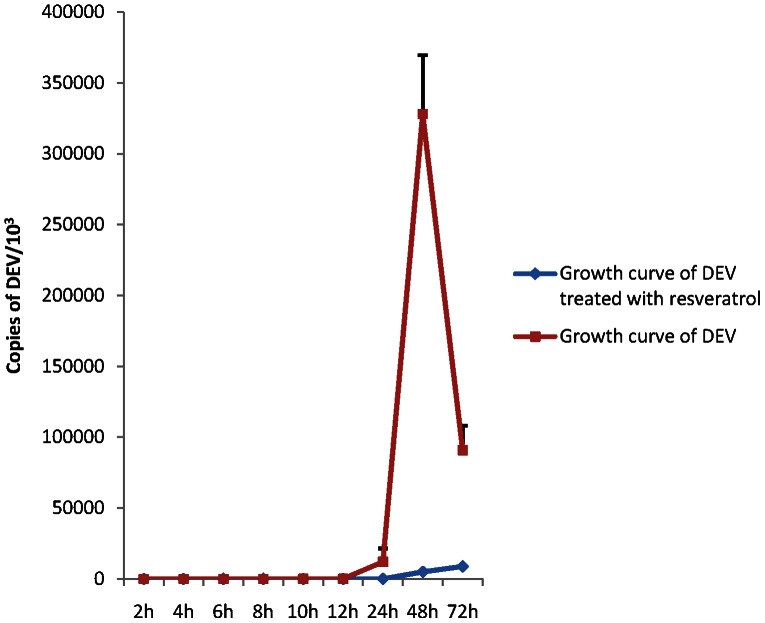
Growth curves of DEV in DEFs in the presence or absence of reveratrol. The DEFs were firstly infected with DEV at MOI of 1 and test resveratrol solution for 1 h at 37°C. DEV proliferated in the DEFs in the presence or absence of reveratrol within 72 h. The total DNA was extracted at the indicated time points (2, 4, 6, 8, 10, 12, 24, 48, and 72 h p.i.) and copies of DEV were detected via real-time FQ-PCR assay. Copies of DEV were calculated according to the standard curve equations: *C*t = −3.328×lg [virus copies/5.25]+40.138 (R^2^ = 0.997). Values are means ± SD (n = 3).

The growth curves of DEV in DEFs in the presence or absence of reveratrol are shown in Fig. 3. Almost no virus proliferation was observed between 0 to 12 h p.i.. DEV began to reproduce at 12 h p.i., and the number of DNA copies showed a rapid increase within the next 24 h. Copies of DEV decreased at 72 h p.i., which is probable due to the nutrient depletion and cell death caused by the large amounts of virus. In the reveratrol group, no increase was observed within 24 h, and the number of copies slowly increased in the next 2 d, which is significantly less than the virus control group without any drug treatment.

### Mode of action

**Figure 4:**
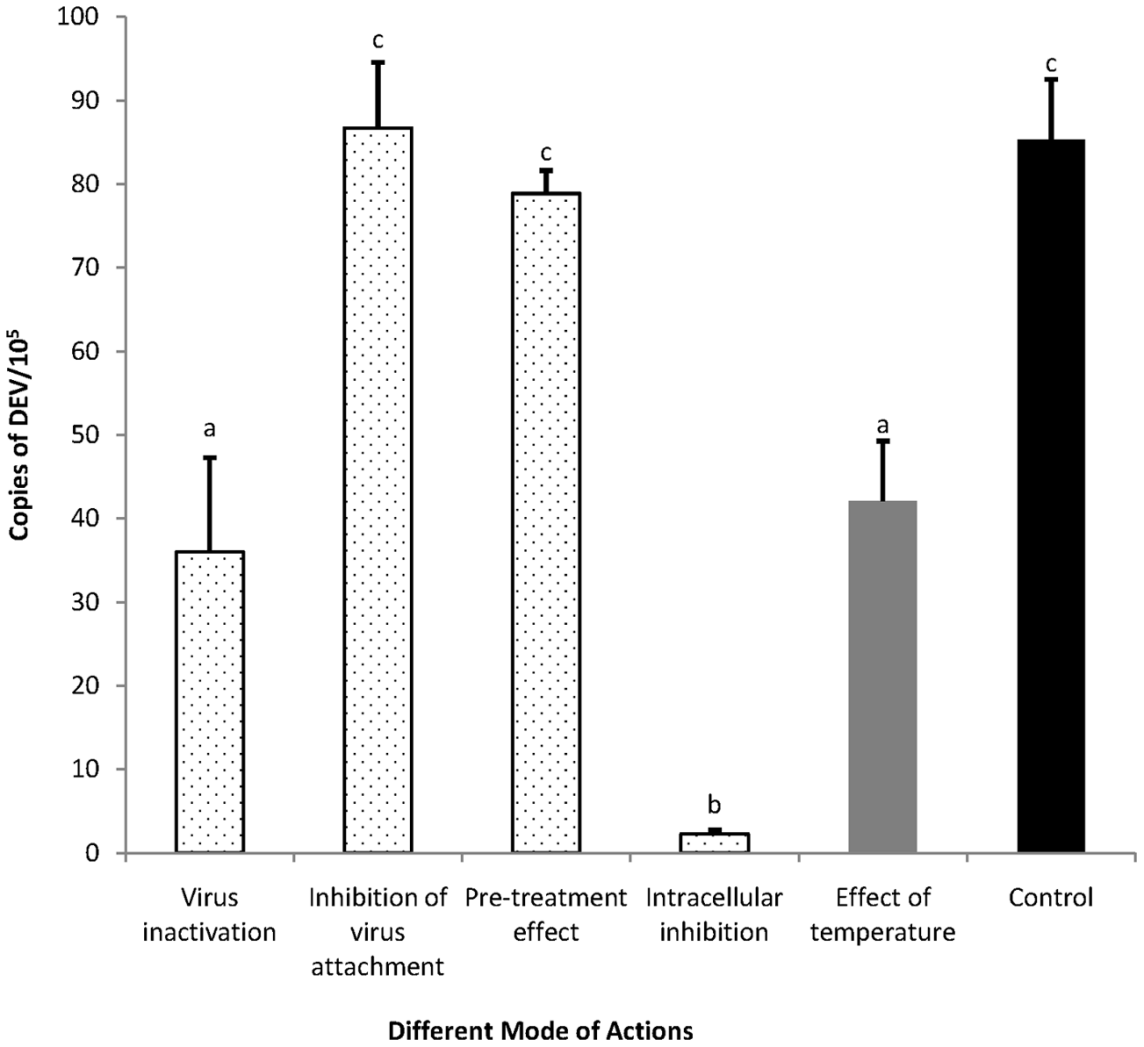
Influence of different treatment conditions of resveratrol on DEV infection. DEFs infected with DEV (MOI  = 0.1) were treated with resveratrol in various mode of actions. (1) Virus inactivation: DEV was treated with drug for 1 h at 37°C before infection. The virus and drug mixture was then added to cells for 1 h at 37°C. The media was removed and replaced with drug-free media. DEV suspended without drug was used as control (effect of temperature). (2) Inhibition of virus attachment: DEFs were infected in media containing the drug and virus for 1 h at 37°C, and then the mixture was removed. The cells were overlaid with drug-free media. (3) Pre-treatment effect: DEFs were pre-treatment with drug for 1 h at 37°C. And then DEV was added to the cells after resveratrol solution removal. After 1 h 37°C, the inoculum was removed and replaced with drug-free media. (4) Intracellular inhibition: after removing the unabsorbed virus, the media containing the resveratrol was added to the DEFs. At 48 h p.i., the total DNA was extracted from DEV-infected DEFs and copies of DEV were evaluated by the real-time FQ-PCR assay. Values are means ± SD (n = 3). Significance: different capital letters represent extremely significant differences among groups (P<0.01, n = 3).

Further study on the mode of action was conducted to evaluate the stage in which resveratrol affects the DEV proliferation cycle. In this assay, 31.25 μg/mL resveratrol was added to the DEFs in various ways, as described above. After incubation for 48 h, total DNA was extracted from DEV-infected DEFs, and copies of DEV were evaluated by the real-time FQ-PCR assay. As shown in Fig. 4, the number of copies of DEV in the group of intracellular inhibition was significantly different from that of the control group (*P*<0.01), whereas in the virus attachment and pre-treatment assays, no significant difference was observed between the test and control groups. The number of copies in the virus inactivation group was also significantly decreased compared with that of the control group. However, it is worth noting that in terms of the effect of temperature assay, which was performed as the control test of virus inactivation assay, the temperature was the key critical element in reducing virus vitality. In other words, the decrease in the number of copies of DEV in the virus inactivation group was mainly due to the incubation for 1 h at 37°C, but not to the treatment with resveratrol.

### Influence of time duration with resveratrol on antiviral activity

A time course study was performed to analyze the influence of addition time of the resveratrol during the virus multiplication intracellular cycle. In this assay, 31.25 μg/mL resveratrol solution was added to the cells after inoculum removal(DEV, 100 TCID_50_) at indicated time points (0, 2, 4, 6, and 8 h p.i.). After incubation for 24 h, cell supernatant was collected, and antiviral activity was evaluated by the virus yield reduction assay.

**Figure 5:**
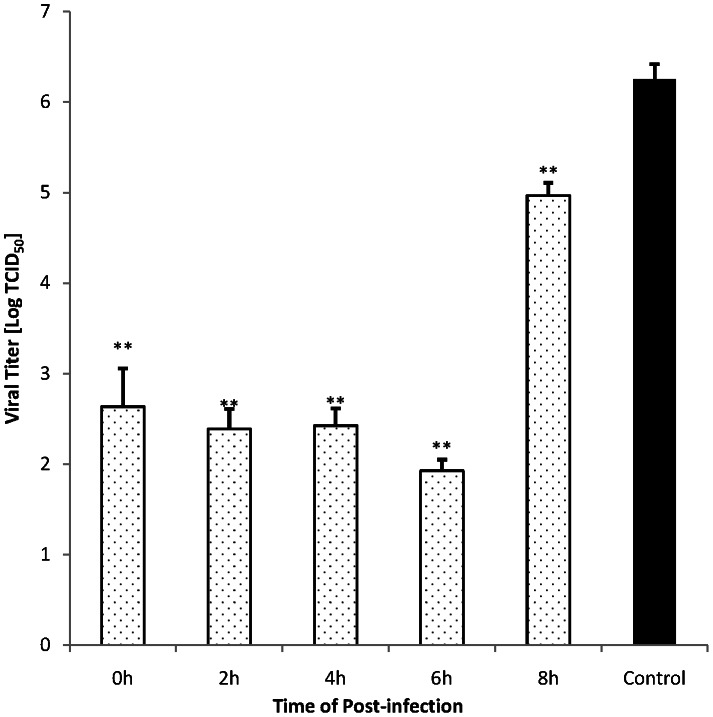
Influence of administration timing of resveratrol on virus infection. DEFs were incubated with DEV (100TCID_50_) for 1 h at 37°C. Resveratrol solution was added to the cells after inoculum removal at indicated time points (0, 2, 4, 6, and 8 h p.i.) at a concentration of 31.25 μg/mL. After incubation for 24 h, antiviral activity was evaluated by the virus yield reduction assay. Values are means ± SD (n = 3). Significance: **P<0.01 vs. non-drug treated control group.

As shown in Fig. 5, when resveratrol was added to DEFs at 0, 2, 4, and 6 h p.i., the viral titers were dramatically decreased compared with the control group. The viral titer also decreased when the administration time was 8 h p.i., but virus vitality was significantly higher than the other administration time. Only a slight difference in the inhibitory effect was observed when resveratrol was added at different p.i. time points within 6 h (0, 2, 4, and 6 h p.i.), and the values of the vitral titers in these groups were equal to the viral titer of inoculums [19]. These results locate the antiviral target of the test drug in an early stage of the virus cycle in the host cells, during the 0–8 h p.i..

### Inhibition of viral replication studied by TEM

**Figure 6:**
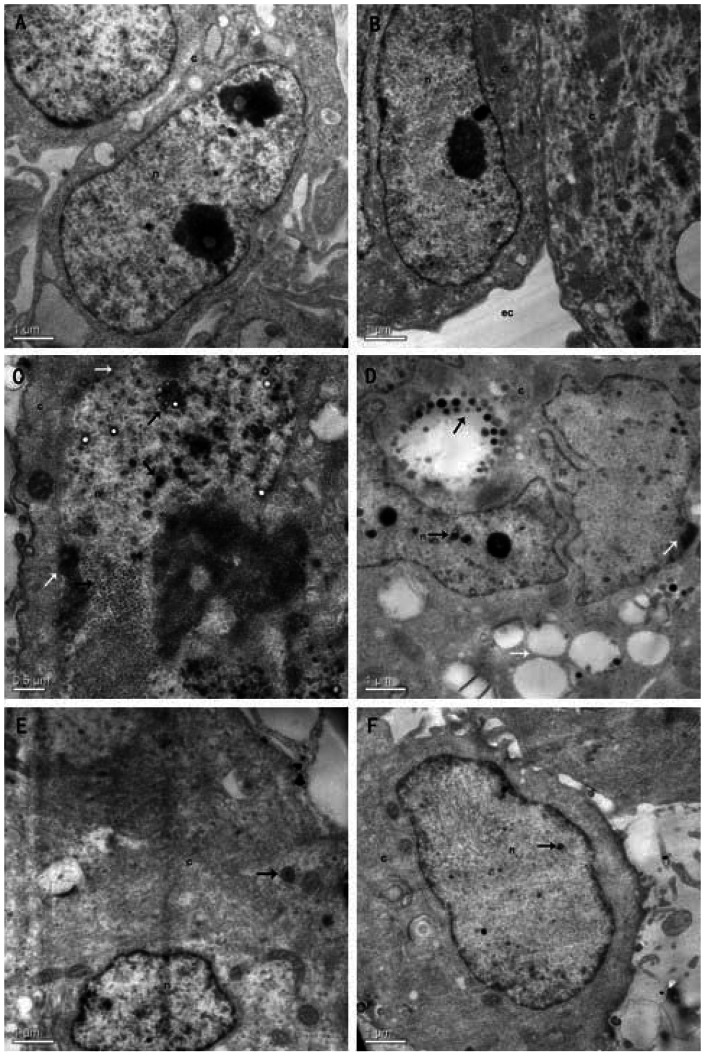
Inhibition of viral replication studied by TEM. DEFs were infected (C, D, E and F, MOI  = 0.1) or infected (A and B) with DEV in the absence (A, B, C and D) or presence (E and F) of 31.25 μg/mL of resveratrol. At 48 h p.i., the replication of DEV in DEFs were studied by TEM. Pictures were taken using a Tecnai G2 F20 electron microscope (FEI, USA). (A and B): normal appearance of cell nucleus and cytoplasm. (C): DEFs infected with DEV. ↗: viral capsids in nucleus; →: a massive accumulation of virus nucleic acids in nucleus; <$>\scale 80%\raster="rg1"<$>: mature viral particles in nucleus; white arrows ↗: chromatin margination; white arrows →: nucleolus membrane burst). (D): DEFs infected with DEV. (↗: Maturating and mature particles in cytoplasmatic vacuoles; →: Maturating particles in nucleus; white arrows ↗: chromatin margination; white arrows →: an extensive vacuoles degeneration). (E and F): Infected DEFs treatment with resveratrol. (→: few viral particles were observed in nucleus and cytoplasm). n: nucleus; c: cytoplasm; ec: extracellular space.

TEM was performed to analyze the effect of resveratrol on vrial replication. DEFs were exposed to resveratrol followed by incubation with DEV (MOI = 0.1) for 1 h at 37°C. 31.25 μg/mL resveratrol was added to the infected cells after inoculums removal. At 48 h p.i., the cells were collected and studied by TEM. Fig. 6A and 6B show the normal appearance of the cell nucleus and cytoplasm. The typical signs of DEV infection of DEFs are as follows: the nucleolus membrane bursts, nucleolus disappears, and chromatin agglutinates with the nuclear membrane; a large number of virus nucleic acids are centrally arranged in the host cell nucleus and the virus capsid gradually forms and waits to be assembled (Fig. 6C); extensive vacuole degeneration occurs and a number of mature particles in cytoplasm vacuoles were observed in Fig. 6D. The application of resveratrol significantly reduced the number of virions in the nucleus and cytoplasm; and nucleoli are not destroyed and few viral particles are observed in the nucleus and cytoplasm (Figs. 6E and 6F).

### Inhibitation on total protein expression

The effect of resveratrol on DEV infection was also monitored via an indirect immunofluorescence assay to determine viral antigen expression. DEFs were exposed to resveratrol followed by incubation with DEV (MOI  = 0.1) for 1 h at 37°C. The infected cells were added with 31.25 μg/mL resveratrol after inoculum removal. At 24 h p.i., antigen expression was detected via immunostaining with anti-DEV antibodies.

**Figure 7:**
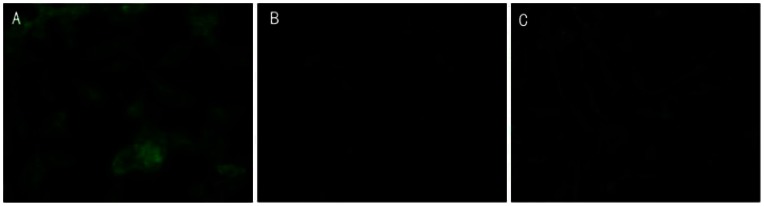
Effect of resveratrol on DEV antigen expression. DEFs were infected with DEV (MOI = 0.1) in the absence (A) or presence (C) of 31.25 μg/mL resveratrol. DEFs without any treatment was used as control (B). At 24 h p.i., immunofluorescence staining was conducted using anti-DEV rabbit antibodies. Magnification: 400×.

The results of a representative experiment are shown in Fig. 7. In DEV-infected cells, a regular dotted distribution of viral proteins in the cell cytoplasm was revealed (Fig. 7A), whereas nearly no fluorescence could be observed in the non-infected cells (Fig. 7B). After treatment with 31.25 μg/mL resveratrol for 24 h, the number of DEV antigen-expressing cells was drastically reduced, and very few sporadic positive cells were detected(Figs. 7C and 7D).

## Discussion

Medicinal plants are increasingly being pursued as suitable alternative sources for discovery of antiviral agents [20],[21],[22],[23]. In China, the long history of medical usage facilitated the investigation of many traditional Chinese herbal medicinesbecause of their antiviral activities, and a growing number of herbs have been determined for experimental and/or clinical antiviral efficacies [24]. In laboratory previous work, we identified the antiviral activities of 18 kinds of traditional Chinese medicines against DEV and discovered that the crude extract of *Rhizoma polygoni cuspidate* can inhibit the infection of DEV in DEFs [13]. Resveratrol, one of the main biologically active ingredients of *Rhizoma polygoni cuspidate* that has been proven to be an efficient agent on several kinds of virus [25],[26],[27],[28],[29],[30],[31],[32],[33],[34], was investigated for its antiviral activity against DEV in this paper. The results indicated that resveratrol effectively reduce the virus titer of DEV in DEFs and protected DEFs from DEV induced cell death in a dose-dependent manner (Fig. 1) with the IC_50_ and SI at 3.85 μg/mL and 15.86 respectively (Table 1). A high inhibitory effect (>75%) was observed when the concentration of resveratrol was 31.25 μg/mL.

Although resveratrol was widely studied for its anti-proliferative mechanisms by inducing reactive oxygen species production and disrupting the release of cytochrome c from the mitochondrial membrane, which leads to cell death in various cancer cell lines [35], references on the precise mechanisms of antiviral activities remain lacking.

In the following experiments, we first detected the influence of resveratrol on virus multiplication in DEFs. Real-time FQ-PCR was performed for this experiment. In traditional antiviral research, the antiviral activities of test samples were confirmed mainly based on their capability to reduce virus titer or inhibit the CPE. Whether the multiplication of virus particles is influenced is unclear. Real-time FQ-PCR based on TaqMan technology, which was performed in this paper, provides an accurate means to quantify viral DNA. Based on this technology, the amount of the virus in cell cultures could be accurately detected at any point in time. In our research, DEV particles began to reproduce 12 h after inoculation and dramatically increased to a peak value at 48 h p.i. (Fig. 3). The growth curve reported in this paper is almost consistent with the result of Guo's [36]. Thus, the influence of resveratrol on the multiplication of DEV in DEFs was analyzed. DNA proliferation was not detected until 24 h p.i. under treatment with resveratrol and the proliferation was maintained at a low level until the end of the test (Fig. 3). This result indicates that resveratrol could inhibit the stage of virus intracellular proliferations. The conclusion of the growth curve assay was further evidenced by the assays for mode of action. In these series of experiments, resveratrol could not inactivate the virus in extracellular and also did not show any significant inhibitory effect during the stage of attachment and entry into the host cell. The inhibitory effect was only observed in the intracellular inhibition assay (Fig. 4).

The stage of virus replication that resveratrol affected was also analyzed. The time of addition study in this paper demonstrated that the resveratrol affected the early stage of the DEV replication cycle (within 8 h p.i.) (Fig. 5). For the alphaherpesvirinae subfamily of herpesviridae, virus replication and package all occur in the host cell's nucleus, in which the genomes of the virus are translated, and some early viral proteins are expressed for further transcription and replication of the viral DNA, then a new virus DNA is synthesized and viral structural proteins are produced; late in the infection, replicated DNAs are packaged into capsids [37]. According to this reference, the early stage of DEV replication might occur in the host cell's nucleus. Thus, we can assume that the process of DEV replication in the host cell's nucleus was inhibited by resveratrol. The TEM photos gave evidence that support the hypothesis that a large number of virus nucleic acids and virus capsids did not observed in the host cell's nucleus compared with the control group (Fig. 6). Similar TEM photos of the DEV infected DEFs were shown in the references [36],[38]. The results of the time of addition assay and the TEM assays locate the antiviral target of the test drug in an early stage of the virus cycle occurs in the host cell's nucleus, which further provide evidence that support the conclusion of the assay on mode of action and further limited the scope of antiviral targets to the host cell's nucleus.

A host nuclear transcription factor activated by HSV infection was suppressed by resveratrol and the synthesis of viral DNA was impaired [39]. A similar result was reported in an antiviral research of resveratrol on VZV where the synthesis of an essential regulatory protein, which is an immediate-early product of viral genome, was negatively impacted by resveratrol, which resulted in the inhibition of VZV replication [31]. Galindo *et al*. reported that resveratrol could inhibit DNA of ASFV replication, late viral protein synthesis and viral factory formation [27]. In our investigation, after incubation for 24 h, protein expression in DEV infected DEFs was effectively inhibited by resveratrol (Fig. 7). According to the experimental results and references, we can assume that resveratrol might have an important function in the regulation of gene transcription and expression to some early proteins that are essential to DEV replication in DEFs.

In conclusion, resveratrol could effectively inhibit DEV in host cells. It does not inactivate the virus in an extracellular manner nor interferes with virus attachment or entry into the host cell directly, but enters into host cells and inhibits the replication of virus nucleic acids and early protein expression in the cell nucleus. The accurate target of resveratrol should be confirmed in further investigations.
